# (Dimethyl­formamide-κ*O*)[2-meth­oxy-6-(2-pyridylmethyl­imino­meth­yl)phenolato-κ^3^ *N*,*N*′,*O* ^1^](thio­cyanato-κ*N*)copper(II)

**DOI:** 10.1107/S1600536810014212

**Published:** 2010-04-24

**Authors:** Qianqian Bao, Xiaodan Chen, Rong Rong, Feifei Shi

**Affiliations:** a Ordered Matter Science Research Center, College of Chemistry and Chemical Engineering, Southeast University, Nanjing 210096, People’s Republic of China; b Department of Chemistry, Key Laboratory of Medicinal Chemistry for Natural Resources, Ministry of Education, Yunnan University, Kunming 650091, People’s Republic of China

## Abstract

In the title compound, [Cu(C_14_H_13_N_2_O_2_)(NCS)(C_3_H_7_NO)], the Cu^2+^ ion is coordinated by an *N*,*N*′,*O*-tridentate 2-meth­oxy-6-(2-pyridylmethyl­imino­meth­yl)phenolate ligand, an *N*-bonded thio­cyanate ion and an *O*-bonded dimethyl­formamide (DMF) mol­ecule, resulting in a distorted CuN_3_O_2_ square-based pyramidal geometry for the metal ion, with the DMF O atom in the apical site. The dihedral angle between the aromatic rings in the ligand is 8.70 (16)°. The S atom is disordered over two positions in a 0.901 (6):0.099 (6) ratio. In the crystal, mol­ecules inter­act by way of π–π stacking inter­actions [centroid–centroid separation = 3.720 (2) Å].

## Related literature

For the synthesis, see: Pointeau *et al.* (1986 ▶). For related structures, see: Li & Zhang (2004 ▶); You & Zhu (2004 ▶).
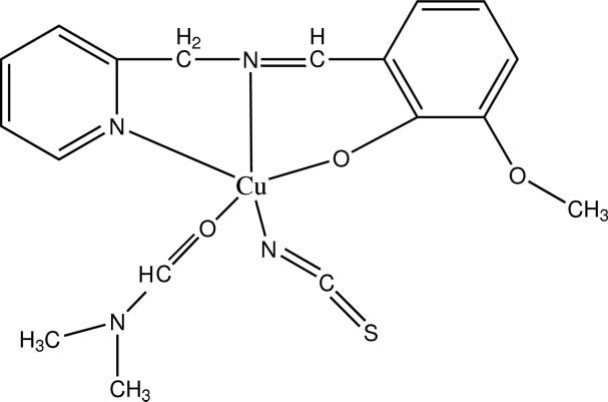


## Experimental

### 

#### Crystal data

[Cu(C_14_H_13_N_2_O_2_)(NCS)(C_3_H_7_NO)]
*M* *_r_* = 435.98
Triclinic, 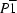

*a* = 8.6768 (9) Å
*b* = 10.9310 (11) Å
*c* = 11.0689 (12) Å
α = 83.251 (2)°
β = 72.023 (1)°
γ = 79.530 (1)°
*V* = 979.84 (18) Å^3^
*Z* = 2
Mo *K*α radiation
μ = 1.25 mm^−1^
*T* = 298 K
0.20 × 0.12 × 0.09 mm

#### Data collection

Rigaku SCXmini diffractometer
Absorption correction: multi-scan (*CrystalClear*; Rigaku, 2005 ▶) *T*_min_ = 0.737, *T*_max_ = 0.868
5129 measured reflections
3392 independent reflections
2679 reflections with *I* > 2σ(*I*)
*R*_int_ = 0.016

#### Refinement

*R*[*F* ^2^ > 2σ(*F* ^2^)] = 0.036
*wR*(*F* ^2^) = 0.090
*S* = 1.09
3392 reflections
251 parameters
H-atom parameters constrained
Δρ_max_ = 0.33 e Å^−3^
Δρ_min_ = −0.37 e Å^−3^

### 

Data collection: *CrystalClear* (Rigaku, 2005 ▶); cell refinement: *CrystalClear*; data reduction: *CrystalClear*; program(s) used to solve structure: *SHELXS97* (Sheldrick, 2008 ▶); program(s) used to refine structure: *SHELXL97* (Sheldrick, 2008 ▶); molecular graphics: *SHELXTL* (Sheldrick, 2008 ▶); software used to prepare material for publication: *SHELXL97*.

## Supplementary Material

Crystal structure: contains datablocks I, global. DOI: 10.1107/S1600536810014212/hb5406sup1.cif

Structure factors: contains datablocks I. DOI: 10.1107/S1600536810014212/hb5406Isup2.hkl

Additional supplementary materials:  crystallographic information; 3D view; checkCIF report

## Figures and Tables

**Table:** 

Cu1—O1	1.905 (2)
Cu1—N1	1.942 (2)
Cu1—N3	1.971 (3)
Cu1—N2	2.012 (2)
Cu1—O3	2.392 (2)

**Table:** 

O1—Cu1—N1	92.88 (9)
O1—Cu1—N3	90.19 (9)
N1—Cu1—N3	168.56 (9)
O1—Cu1—N2	173.84 (9)
N1—Cu1—N2	82.13 (10)
N3—Cu1—N2	94.01 (10)
O1—Cu1—O3	93.96 (8)
N1—Cu1—O3	96.07 (8)
N3—Cu1—O3	94.71 (9)
N2—Cu1—O3	90.18 (8)

**Fig. 1.**
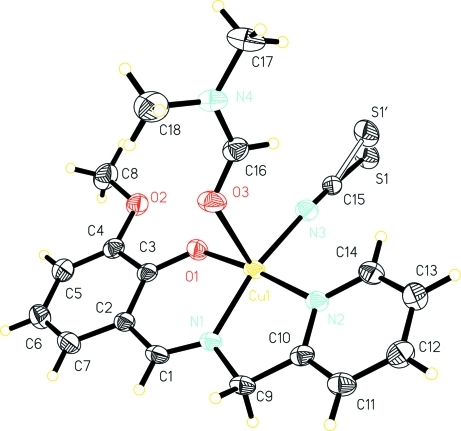
The molecular structure of (I) with displacement ellipsoids drawn at the 30% probability level.

## supplementary crystallographic information

### Experimental

3-Methoxysalicylaldehyde (0.0152 g, 0.1 mmol) dissolved in methanol (5 ml) was added to a methanol solution (5 ml) of 2-aminopyridine (0.0108 g, 0.1 mmol) with slowly stirring. The resulting yellow solution was continuously stirred for about 1 h, then CuCl_2_.2H_2_O (0.017 g, 0.1 mmol) in 5 ml water and potassium thiocyanate (0.019 g, 0.2 mmol) in 2 ml methanol were added with stirring. The precipitate was collected by filtration, dissolved with *N*,*N*-dimethyllformamide. Brown prisms of (I) were obtained by slow evaporation at room temperature over several days.

### Refinement

H atoms bound to carbon were placed in geometrical positions and refined using a riding model, with C—H = 0.94Å and U_iso_(H) =1.2U_eq_(C).

### Crystal data

**Table:**

[Cu(C_14_H_13_N_2_O_2_)(NCS)(C_3_H_7_NO)]	*Z* = 2
*M**_r_* = 435.98	*F*(000) = 450
Triclinic, *P*1	*D*_x_ = 1.478 Mg m^−^^3^
Hall symbol: -P 1	Mo *K*α radiation, λ = 0.71073 Å
*a* = 8.6768 (9) Å	Cell parameters from 13380 reflections
*b* = 10.9310 (11) Å	θ = 3.0–27.6°
*c* = 11.0689 (12) Å	µ = 1.25 mm^−^^1^
α = 83.251 (2)°	*T* = 298 K
β = 72.023 (1)°	Prism, dark brown
γ = 79.530 (1)°	0.20 × 0.12 × 0.09 mm
*V* = 979.84 (18) Å^3^	

### Data collection

**Table:**

Rigaku SCXmini diffractometer	3392 independent reflections
Radiation source: fine-focus sealed tube	2679 reflections with *I* > 2σ(*I*)
graphite	*R*_int_ = 0.016
Detector resolution: 8.192 pixels mm^-1^	θ_max_ = 25.0°, θ_min_ = 1.9°
Thin–slice ω scans	*h* = −9→10
Absorption correction: multi-scan (*CrystalClear*; Rigaku, 2005)	*k* = −13→7
*T*_min_ = 0.737, *T*_max_ = 0.868	*l* = −12→13
5129 measured reflections	

### Refinement

**Table:**

Refinement on *F*^2^	Primary atom site location: structure-invariant direct methods
Least-squares matrix: full	Secondary atom site location: difference Fourier map
*R*[*F*^2^ > 2σ(*F*^2^)] = 0.036	Hydrogen site location: inferred from neighbouring sites
*wR*(*F*^2^) = 0.090	H-atom parameters constrained
*S* = 1.09	*w* = 1/[σ^2^(*F*_o_^2^) + (0.039*P*)^2^ + 0.2715*P*] where *P* = (*F*_o_^2^ + 2*F*_c_^2^)/3
3392 reflections	(Δ/σ)_max_ = 0.001
251 parameters	Δρ_max_ = 0.33 e Å^−^^3^
0 restraints	Δρ_min_ = −0.37 e Å^−^^3^

### Special details

**Table:**

Geometry. All esds (except the esd in the dihedral angle between two l.s. planes) are estimated using the full covariance matrix. The cell esds are taken into account individually in the estimation of esds in distances, angles and torsion angles; correlations between esds in cell parameters are only used when they are defined by crystal symmetry. An approximate (isotropic) treatment of cell esds is used for estimating esds involving l.s. planes.
Refinement. Refinement of *F*^2^ against ALL reflections. The weighted *R*-factor wR and goodness of fit *S* are based on *F*^2^, conventional *R*-factors *R* are based on *F*, with *F* set to zero for negative *F*^2^. The threshold expression of *F*^2^ > σ(*F*^2^) is used only for calculating *R*-factors(gt) etc. and is not relevant to the choice of reflections for refinement. *R*-factors based on *F*^2^ are statistically about twice as large as those based on *F*, and *R*- factors based on ALL data will be even larger.

### Fractional atomic coordinates and isotropic or equivalent isotropic displacement parameters (Å^2^)

**Table:**

	*x*	*y*	*z*	*U*_iso_*/*U*_eq_	Occ. (<1)
Cu1	0.63382 (4)	0.88140 (3)	0.58156 (3)	0.03823 (14)	
S1	0.1914 (3)	0.73555 (19)	0.89276 (15)	0.0641 (6)	0.901 (6)
S1'	0.255 (2)	0.6960 (16)	0.9198 (14)	0.0641 (6)	0.099 (6)
N1	0.7507 (3)	0.9830 (2)	0.4385 (2)	0.0367 (5)	
N2	0.6888 (3)	1.0010 (2)	0.6814 (2)	0.0386 (6)	
N3	0.4812 (3)	0.8079 (2)	0.7321 (2)	0.0450 (6)	
N4	0.9692 (3)	0.5684 (2)	0.7084 (3)	0.0512 (7)	
O1	0.5788 (2)	0.78300 (18)	0.47433 (19)	0.0442 (5)	
O2	0.4899 (3)	0.61550 (19)	0.3729 (2)	0.0514 (6)	
O3	0.8691 (3)	0.73557 (19)	0.5981 (2)	0.0545 (6)	
C1	0.7883 (3)	0.9606 (3)	0.3205 (3)	0.0405 (7)	
H1	0.8535	1.0123	0.2618	0.049*	
C2	0.7394 (3)	0.8639 (3)	0.2710 (3)	0.0385 (7)	
C3	0.6353 (3)	0.7818 (3)	0.3508 (3)	0.0385 (7)	
C4	0.5880 (4)	0.6919 (3)	0.2902 (3)	0.0430 (7)	
C5	0.6418 (4)	0.6871 (3)	0.1600 (3)	0.0514 (8)	
H5	0.6084	0.6291	0.1223	0.062*	
C6	0.7457 (4)	0.7678 (3)	0.0834 (3)	0.0575 (9)	
H6	0.7818	0.7628	−0.0044	0.069*	
C7	0.7939 (4)	0.8537 (3)	0.1377 (3)	0.0507 (8)	
H7	0.8639	0.9066	0.0863	0.061*	
C8	0.4532 (5)	0.5136 (3)	0.3227 (4)	0.0697 (11)	
H8A	0.3792	0.5445	0.2732	0.105*	
H8B	0.4032	0.4579	0.3914	0.105*	
H8C	0.5526	0.4698	0.2696	0.105*	
C9	0.8024 (4)	1.0923 (3)	0.4699 (3)	0.0417 (7)	
H9A	0.7397	1.1677	0.4433	0.050*	
H9B	0.9175	1.0936	0.4252	0.050*	
C10	0.7757 (3)	1.0878 (3)	0.6111 (3)	0.0388 (7)	
C11	0.8352 (4)	1.1694 (3)	0.6660 (3)	0.0479 (8)	
H11	0.8973	1.2277	0.6155	0.058*	
C12	0.8012 (4)	1.1628 (3)	0.7959 (3)	0.0525 (8)	
H12	0.8393	1.2173	0.8345	0.063*	
C13	0.7099 (4)	1.0746 (3)	0.8691 (3)	0.0550 (9)	
H13	0.6850	1.0692	0.9573	0.066*	
C14	0.6569 (4)	0.9956 (3)	0.8089 (3)	0.0496 (8)	
H14	0.5964	0.9356	0.8579	0.059*	
C15	0.3643 (4)	0.7751 (3)	0.7999 (3)	0.0399 (7)	
C16	0.8581 (4)	0.6634 (3)	0.6928 (3)	0.0490 (8)	
H16	0.7623	0.6769	0.7599	0.059*	
C17	0.9490 (5)	0.4915 (4)	0.8262 (4)	0.0790 (12)	
H17A	1.0281	0.5039	0.8662	0.118*	
H17B	0.9649	0.4055	0.8087	0.118*	
H17C	0.8405	0.5142	0.8820	0.118*	
C18	1.1236 (5)	0.5440 (4)	0.6098 (4)	0.0901 (14)	
H18A	1.1135	0.5866	0.5310	0.135*	
H18B	1.1522	0.4559	0.5996	0.135*	
H18C	1.2077	0.5731	0.6333	0.135*	

### Atomic displacement parameters (Å^2^)

**Table:**

	*U*^11^	*U*^22^	*U*^33^	*U*^12^	*U*^13^	*U*^23^
Cu1	0.0348 (2)	0.0348 (2)	0.0399 (2)	−0.00972 (15)	−0.00338 (15)	0.00433 (15)
S1	0.0534 (10)	0.0732 (10)	0.0568 (7)	−0.0317 (8)	0.0074 (7)	0.0010 (6)
S1'	0.0534 (10)	0.0732 (10)	0.0568 (7)	−0.0317 (8)	0.0074 (7)	0.0010 (6)
N1	0.0332 (13)	0.0317 (13)	0.0418 (15)	−0.0070 (10)	−0.0076 (11)	0.0055 (10)
N2	0.0333 (13)	0.0359 (14)	0.0412 (15)	−0.0073 (10)	−0.0041 (11)	0.0034 (11)
N3	0.0414 (14)	0.0411 (15)	0.0473 (16)	−0.0123 (12)	−0.0039 (13)	0.0023 (12)
N4	0.0492 (16)	0.0414 (16)	0.0636 (18)	−0.0062 (13)	−0.0228 (14)	0.0096 (13)
O1	0.0451 (12)	0.0429 (12)	0.0410 (13)	−0.0173 (9)	−0.0029 (10)	0.0004 (9)
O2	0.0515 (13)	0.0434 (13)	0.0574 (14)	−0.0159 (10)	−0.0072 (11)	−0.0065 (10)
O3	0.0445 (13)	0.0485 (14)	0.0601 (15)	0.0008 (10)	−0.0118 (11)	0.0156 (11)
C1	0.0311 (15)	0.0377 (17)	0.0450 (19)	−0.0043 (13)	−0.0058 (13)	0.0120 (13)
C2	0.0321 (15)	0.0351 (16)	0.0425 (18)	−0.0011 (12)	−0.0070 (13)	0.0034 (13)
C3	0.0310 (15)	0.0325 (16)	0.0461 (19)	0.0040 (12)	−0.0092 (13)	0.0006 (13)
C4	0.0359 (16)	0.0380 (17)	0.052 (2)	−0.0006 (13)	−0.0120 (14)	−0.0023 (14)
C5	0.052 (2)	0.050 (2)	0.054 (2)	−0.0060 (16)	−0.0167 (17)	−0.0080 (16)
C6	0.065 (2)	0.063 (2)	0.0405 (19)	−0.0064 (18)	−0.0121 (17)	−0.0028 (16)
C7	0.0478 (19)	0.054 (2)	0.0447 (19)	−0.0083 (15)	−0.0085 (15)	0.0073 (15)
C8	0.071 (2)	0.055 (2)	0.084 (3)	−0.0228 (19)	−0.012 (2)	−0.021 (2)
C9	0.0393 (16)	0.0373 (17)	0.0471 (19)	−0.0141 (13)	−0.0109 (14)	0.0100 (13)
C10	0.0282 (14)	0.0326 (16)	0.0502 (19)	−0.0010 (12)	−0.0074 (13)	0.0014 (13)
C11	0.0413 (17)	0.0449 (19)	0.056 (2)	−0.0119 (14)	−0.0129 (15)	0.0064 (15)
C12	0.052 (2)	0.050 (2)	0.060 (2)	−0.0109 (16)	−0.0202 (17)	−0.0063 (16)
C13	0.059 (2)	0.058 (2)	0.046 (2)	−0.0076 (17)	−0.0131 (17)	−0.0038 (16)
C14	0.0510 (19)	0.0473 (19)	0.047 (2)	−0.0154 (15)	−0.0074 (16)	0.0042 (15)
C15	0.0478 (18)	0.0331 (16)	0.0367 (17)	−0.0109 (14)	−0.0066 (15)	−0.0022 (13)
C16	0.0421 (18)	0.0425 (19)	0.059 (2)	−0.0060 (15)	−0.0104 (16)	−0.0016 (16)
C17	0.090 (3)	0.068 (3)	0.080 (3)	−0.016 (2)	−0.039 (2)	0.032 (2)
C18	0.061 (2)	0.090 (3)	0.092 (3)	0.024 (2)	−0.012 (2)	0.016 (2)

### Geometric parameters (Å, °)

**Table:**

Cu1—O1	1.905 (2)	C5—H5	0.9300
Cu1—N1	1.942 (2)	C6—C7	1.358 (5)
Cu1—N3	1.971 (3)	C6—H6	0.9300
Cu1—N2	2.012 (2)	C7—H7	0.9300
Cu1—O3	2.392 (2)	C8—H8A	0.9600
S1—C15	1.635 (3)	C8—H8B	0.9600
S1'—C15	1.633 (13)	C8—H8C	0.9600
N1—C1	1.287 (4)	C9—C10	1.504 (4)
N1—C9	1.463 (3)	C9—H9A	0.9700
N2—C10	1.342 (4)	C9—H9B	0.9700
N2—C14	1.347 (4)	C10—C11	1.385 (4)
N3—C15	1.149 (4)	C11—C12	1.372 (4)
N4—C16	1.317 (4)	C11—H11	0.9300
N4—C18	1.447 (5)	C12—C13	1.383 (4)
N4—C17	1.447 (4)	C12—H12	0.9300
O1—C3	1.304 (3)	C13—C14	1.368 (4)
O2—C4	1.364 (4)	C13—H13	0.9300
O2—C8	1.426 (4)	C14—H14	0.9300
O3—C16	1.225 (4)	C16—H16	0.9300
C1—C2	1.427 (4)	C17—H17A	0.9600
C1—H1	0.9300	C17—H17B	0.9600
C2—C7	1.416 (4)	C17—H17C	0.9600
C2—C3	1.420 (4)	C18—H18A	0.9600
C3—C4	1.433 (4)	C18—H18B	0.9600
C4—C5	1.375 (4)	C18—H18C	0.9600
C5—C6	1.397 (5)		
			
O1—Cu1—N1	92.88 (9)	O2—C8—H8B	109.5
O1—Cu1—N3	90.19 (9)	H8A—C8—H8B	109.5
N1—Cu1—N3	168.56 (9)	O2—C8—H8C	109.5
O1—Cu1—N2	173.84 (9)	H8A—C8—H8C	109.5
N1—Cu1—N2	82.13 (10)	H8B—C8—H8C	109.5
N3—Cu1—N2	94.01 (10)	N1—C9—C10	109.5 (2)
O1—Cu1—O3	93.96 (8)	N1—C9—H9A	109.8
N1—Cu1—O3	96.07 (8)	C10—C9—H9A	109.8
N3—Cu1—O3	94.71 (9)	N1—C9—H9B	109.8
N2—Cu1—O3	90.18 (8)	C10—C9—H9B	109.8
C1—N1—C9	118.2 (2)	H9A—C9—H9B	108.2
C1—N1—Cu1	125.6 (2)	N2—C10—C11	121.8 (3)
C9—N1—Cu1	116.20 (18)	N2—C10—C9	116.1 (3)
C10—N2—C14	118.4 (3)	C11—C10—C9	122.2 (3)
C10—N2—Cu1	115.2 (2)	C12—C11—C10	119.0 (3)
C14—N2—Cu1	126.2 (2)	C12—C11—H11	120.5
C15—N3—Cu1	161.4 (3)	C10—C11—H11	120.5
C16—N4—C18	120.1 (3)	C11—C12—C13	119.5 (3)
C16—N4—C17	122.3 (3)	C11—C12—H12	120.2
C18—N4—C17	117.5 (3)	C13—C12—H12	120.2
C3—O1—Cu1	127.30 (19)	C14—C13—C12	118.5 (3)
C4—O2—C8	117.7 (3)	C14—C13—H13	120.7
C16—O3—Cu1	119.2 (2)	C12—C13—H13	120.7
N1—C1—C2	126.2 (3)	N2—C14—C13	122.7 (3)
N1—C1—H1	116.9	N2—C14—H14	118.6
C2—C1—H1	116.9	C13—C14—H14	118.6
C7—C2—C3	119.9 (3)	N3—C15—S1'	156.9 (9)
C7—C2—C1	118.0 (3)	N3—C15—S1	176.5 (3)
C3—C2—C1	122.1 (3)	S1'—C15—S1	26.5 (8)
O1—C3—C2	124.9 (3)	O3—C16—N4	126.1 (3)
O1—C3—C4	117.8 (3)	O3—C16—H16	116.9
C2—C3—C4	117.3 (3)	N4—C16—H16	116.9
O2—C4—C5	125.5 (3)	N4—C17—H17A	109.5
O2—C4—C3	113.9 (3)	N4—C17—H17B	109.5
C5—C4—C3	120.6 (3)	H17A—C17—H17B	109.5
C4—C5—C6	121.2 (3)	N4—C17—H17C	109.5
C4—C5—H5	119.4	H17A—C17—H17C	109.5
C6—C5—H5	119.4	H17B—C17—H17C	109.5
C7—C6—C5	119.8 (3)	N4—C18—H18A	109.5
C7—C6—H6	120.1	N4—C18—H18B	109.5
C5—C6—H6	120.1	H18A—C18—H18B	109.5
C6—C7—C2	121.2 (3)	N4—C18—H18C	109.5
C6—C7—H7	119.4	H18A—C18—H18C	109.5
C2—C7—H7	119.4	H18B—C18—H18C	109.5
O2—C8—H8A	109.5		
